# Responsiveness of PROMIS® to change in chronic obstructive pulmonary disease

**DOI:** 10.1186/s41687-019-0155-9

**Published:** 2019-10-29

**Authors:** Susan E. Yount, Charles Atwood, James Donohue, Ron D. Hays, Debra Irwin, Nancy Kline Leidy, Honghu Liu, Karen L. Spritzer, Darren A. DeWalt

**Affiliations:** 1 Department of Medical Social Sciences, Northwestern University Feinberg School of Medicine, 625 N. Michigan Avenue, 27th floor, Chicago, IL 60611 USA; 2 Pulmonary Section, VA Pittsburgh Healthcare System and University of Pittsburgh Medical Center, Pittsburgh, PA USA; 3 Division of Pulmonary Medicine, University of North Carolina School of Medicine, University of North Carolina, Chapel Hill, NC USA; 4 Department of Medicine, University of California, Los Angeles, Los Angeles, CA USA; 5 Department of Epidemiology, Gillings School of Global Public Health, University of North Carolina – Chapel Hill, Chapel Hill, NC USA; 6 Evidera, Bethesda, MD USA; 7 School of Dentistry, University of California, Los Angeles, Los Angeles, CA USA; 8 Division of General Medicine and Clinical Epidemiology and the Cecil G. Sheps Center for Health Services Research, University of North Carolina School of Medicine – Chapel Hill, Chapel Hill, NC USA

**Keywords:** Chronic obstructive pulmonary disease, COPD, Patient-reported outcomes, PROMIS

## Abstract

**Background:**

Chronic obstructive pulmonary disease (COPD) is a progressive chronic disease characterized by airflow obstruction that leads to shortness of breath and substantial negative impacts on health-related quality of life (HRQL). The course of COPD includes periodic acute exacerbations that require changes in treatment and/or hospitalizations. This study was designed to examine the responsiveness of Patient-Reported Outcomes Measurement Information System® (PROMIS®) measures to changes associated with COPD exacerbation recovery.

**Methods:**

A longitudinal analysis using mixed-effects models was conducted of people who were enrolled while stable (*n* = 100) and those who experienced an acute exacerbation (*n* = 85). PROMIS (physical function, pain interference, pain behavior, fatigue, anxiety, depression, anger, social roles, discretionary social activities, Global Health, dyspnea severity and dyspnea functional limitations) and COPD-targeted HRQL measures were completed at baseline and at 12 weeks.

**Results:**

We administered PROMIS measures using computer adaptive testing (CAT), followed by administration of any remaining short form (SF) items that had not yet been administered by CAT. Examination of the difference between group differences from baseline to 12 weeks in the stable and exacerbation groups revealed that the exacerbation group changed (improved) significantly more than the stable group in anxiety (*p* < .001 to *p <* .01; f^2^ effect size [ES] = 0.023/0.021), fatigue (*p* < .0001; ES = 0.036/0.047) and social roles (*p* < .001 to *p <* .05; ES = 0.035/0.024). All effect sizes were small in magnitude and smaller than hypothesized. Depression was also statistically significant (*p* < .05, SF only) but the ES was trivial. For all other PROMIS domains, the differences were not significant and ES were trivial.

**Conclusions:**

This longitudinal study provides some support for the validity of the PROMIS fatigue, anxiety, and social roles domains in COPD, but further evaluation of responsiveness is warranted.

## Background

Chronic obstructive pulmonary disease (COPD) is a heterogeneous group of slowly progressive diseases characterized by airflow obstruction that interferes with normal breathing and leads to shortness of breath or dyspnea that can limit physical activity [1]. COPD is the third leading cause of death in the U.S. and the only leading cause of death increasing in prevalence [2, 3]. Those with COPD experience limitations in functioning and well-being or health-related quality of life (HRQL) comparable to or worse than patients with advanced lung cancer [4].

COPD leads to progressive decline in lung function associated with worsening of symptoms. Many patients experience periodic exacerbations, defined as an acute sustained worsening of their COPD, that result in unscheduled clinic or emergency department (ED) visits and require antibiotics and/or steroids, with severe cases requiring hospitalization for observation and treatment. The decline in lung function, symptoms, and physical function associated with exacerbations represent substantial negative impacts on HRQL and contribute to the downward trajectory of disease over time [5, 6].

Measures of lung function, such as forced expiratory volume in 1 s (FEV_1_) and forced vital capacity (FVC), are used to estimate the severity of airflow limitation but fail to capture the systemic manifestations and patient-experienced impact of the disease [7, 8]. Hence, patient-reported outcomes (PROs) are needed in COPD observational studies, clinical trials, and patient care [9]. Stable COPD is associated with relatively poor status in several PRO domains, namely depression [10, 11], anxiety [11], fatigue [12–15], mobility, activities of daily living (ADLs) [13] and social activities [13]. Acute exacerbations of COPD are, by definition, characterized by worsening of respiratory symptoms, including dyspnea [16–19] as well as decreased health-related quality of life [20–22], lower levels of physical function [5, 20, 23, 24]; increased fatigue [23–26], depression and anxiety [23, 27–31], reduced social functioning [23, 28], and to a lesser degree, increased pain [23] and anger [23]. Thus, COPD is a potentially informative target condition for evaluating the measurement properties of PROs, including change in outcomes that are expected to change with recovery from an exacerbation (e.g., fatigue, anxiety, mobility, ADLs, social activities) and stability in outcomes that are expected to remain relatively unchanged (e.g., depression).

The Patient Reported Outcomes Measurement Information System® (PROMIS®) quantifies self-reported health with domains relevant to multiple chronic diseases and conditions, permitting selective or comprehensive outcome assessment based on user interests and needs. Each measure comprising this system was developed using standardized, rigorous psychometric methods [32, 33] with testing in the general population and across a number of different patient subgroups [34–39]. Most PROMIS measures are universal (i.e., not disease-specific), but some are particularly relevant to patients with COPD such as fatigue, physical function, anxiety, depression, dyspnea and social function. Although other instruments exist to measure some or all of these outcomes in various combinations [7, 40], PROMIS offers single-site open access (www.healthmeasures.net) to each measure, computer adaptive test (CAT) versions for efficient assessments, and information on normative values. Cross-sectional analyses in patients with COPD provide support for the reliability and validity of PROMIS measures [41].

Acute exacerbations of COPD are clinically relevant events with important therapeutic and prognostic implications and with considerable heterogeneity in terms of their clinical presentation [42]. The majority of the literature on impacts of acute exacerbations of COPD has focused on respiratory symptoms, morbidity, mortality, hospitalizations and disease-specific health-related quality of life questionnaires (e.g., St. George’s Respiratory Questionnaire [43]) [44–51]. In contrast, this study examines the responsiveness of PROMIS measures of specific physical, mental and social health status domains over 12 weeks in patients with COPD who were recovering from an acute COPD exacerbation. We also studied patients with COPD who were enrolled in a stable (non-exacerbating) state and followed for 12 weeks to allow us to explore any changes in the measures that were unrelated to exacerbation recovery. We hypothesized that there would be significant improvement in most of the PROMIS domain measures in COPD patients during recovery from exacerbation to a stable period, and that the largest magnitude changes would be in physical function, fatigue, anxiety, dyspnea and social function. While those in the stable condition might change over time due to unmeasured factors, we hypothesized that there would be little to no change over 12 weeks, and any observed change would be smaller than that of patients recovering from an acute exacerbation.

## Methods

This study used a longitudinal, multisite prospective cohort of two groups: 1) patients with COPD enrolled at the time of an exacerbation, and 2) patients with COPD enrolled at a time of stability. Both groups were followed for 12 weeks and completed assessments at enrollment (baseline) and then weekly for the remaining 11 weeks. In this paper, we report on the baseline and 12-week findings. Subjects were recruited from outpatient clinics and hospitals at four research sites (University of North Carolina Health System, NorthShore University HealthSystem, Pittsburgh VA Medical Center, and Durham VA Medical Center).

### Participants

We enrolled patients 40 years and older with an established clinical history of COPD in accordance with the GOLD definition at the time of the study [52] and at least a 10 pack/year history of smoking. Participants had to be able to read and speak English and be able to see and interact with a computer screen, mouse, and keyboard. People were excluded if, based on the input of clinic staff or evidence in the medical chart, they had a concurrent medical or psychiatric condition that precluded participation in the study or completion of self-report questionnaires (e.g., dementia, uncontrolled schizophrenia). They were also excluded if they had a history of asthma without co-existent COPD or were experiencing a heart failure exacerbation. For those enrolled into the exacerbation group, participants recruited in the outpatient setting may have started treatment no more than 3 days prior to the day of enrollment, and for participants recruited in the inpatient setting, no more than 6 days prior to the day of enrollment. Those enrolled into the stable state group had to be exacerbation-free for a minimum of 2 months prior to enrollment. An exacerbation was defined as sustained worsening of COPD symptoms from stable state and beyond normal day-to-day variations that is acute in onset and necessitates a change in regular medication in a patient with underlying COPD; in addition, the exacerbation had to be established by a clinic visit or hospitalization with a medical diagnosis of COPD exacerbation and treatment with antibiotics or corticosteroids [53].

The study was conducted in accordance with the amended Declaration of Helsinki and was approved by the Institutional Review Board (IRB) at each site (University of North Carolina, 08–0138; NorthShore University HealthSystem, EH04–179; Pittsburgh VA Medical Center, 02683; Duke University, Pro00006904). At the time of enrollment and prior to beginning the baseline assessment, informed consent was obtained from all participants included in the study.

### Procedures

For those stable at enrollment, the baseline assessment included questionnaire measures, pulmonary function testing with GOLD classification [54], and a six-minute walk test. Because of the compromised health of those in an exacerbation state at enrollment, only the questionnaires were administered; the six-minute walk test (6MWT) and pulmonary function testing with GOLD classification were performed at the 12-week follow-up when patients had returned to stable state. Thus, all analysis of clinical measures (i.e., pulmonary function testing, GOLD classification, 6MWT) reflected data obtained when patients were deemed stable.

All baseline questionnaires were completed by patients on a laptop computer in the clinic or in the hospital and included demographics, comorbid conditions, COPD history (symptoms, duration of diagnosis, number of exacerbations, and recent hospitalizations and ED visits), and the PRO measures. The research assistant also reviewed the clinical chart to record clinical variables such as body mass index (BMI) and COPD medications. If patients completed pulmonary function tests in-clinic that same day, the values were obtained from the medical chart and they were not asked to repeat the spirometry for the study. The follow-up at 12 weeks (+ 30-day window) was completed in-person in the clinic on the laptop computer. If, during the course of follow-up, a participant had a recurrent exacerbation, as defined previously, they were censored from analysis.

### Measures

#### PROMIS measures

The primary goal of this study was to assess the responsiveness of PROMIS version 1.0 measures to changes associated with COPD exacerbation recovery. These measures assessed anxiety, depression, anger [55], fatigue [56], pain behavior [57], pain interference [58], physical function [59], satisfaction with participation in discretionary social activities [discretionary social activities], satisfaction with participation in social roles [social roles] [60], the 10-item Global Health short form [61] (producing scores for physical health and mental health) and dyspnea (Functional Assessment of Chronic Illness Therapy (FACIT) dyspnea severity and functional limitations measures). The FACIT dyspnea short forms (now also included as PROMIS measures) consist of items that assess dyspnea severity (10 items) and related functional limitations (10 items) and were newly developed, with this being the first longitudinal administration [62, 63].

PROMIS measures can be administered as fixed-length short forms (SF) or dynamically by CAT. Questionnaires were administered using Assessment Center^SM^, a web-based data collection platform [64]. For PROMIS CATs, the first item administered in a CAT is usually one in the middle of the range of function or symptom severity. After a person provides a response, an estimated score is calculated. The CAT algorithm then selects the best item in the item bank for refining the estimated score. After a person provides a response, the estimated score is recalculated. The CAT continues to administer items until a specified level of measurement precision is reached (standard error < 0.3 on theta metric or 3.0 on a T-score metric) or a specified maximum number of items was administered (12). For this study, Assessment Center administered the PROMIS measures using CAT, followed by administration of any remaining SF items that had not yet been administered by CAT. These SF items were derived from the version 1.0 anger 8a, anxiety 7a, depression 8b, fatigue 7a, physical function 10a, pain interference 6b, pain behavior 7a, discretionary social activities 7a, and social roles 7a short forms.

PROMIS scores are estimated using item response theory parameters and scored on a T-score metric, with 50 typically representing the mean and 10 the standard deviation in the U. S. general population (for most domains). Exceptions to this include the PROMIS dyspnea severity and functional limitations measures, for which the mean and standard deviation (50/10) reflect the sample on which the measures were developed - people with COPD. For all PROMIS measures, the direction of scoring is guided by the domain name; higher scores indicate more of the construct being measured. Thus, for the PROMIS domains of anger, anxiety, depression, fatigue, dyspnea severity, dyspnea functional limitations, pain behavior and pain interference, higher scores indicate worse health, and for domains of physical function, discretionary social activities, social roles, and Global Health (physical health and mental health), higher scores indicate better health.

#### Additional measures

We administered two PRO assessments commonly used to assess COPD or related symptoms at baseline and week 12. The St. George's Respiratory Questionnaire (SGRQ) contains three domains (symptoms, activity, and impacts) and a summary score on a 0–100 possible range with 100 representing the worst HRQL [43]. The Modified Medical Research Council (MMRC) dyspnea scale is scored on a scale of 0 to 4 (0 = not troubled with breathlessness except with strenuous exercise, to 4 = too breathless to leave the house or breathless when dressing or undressing) [65, 66]. We transformed the MMRC score linearly to a 0–100 possible range (1 = 0; 2 = 25; 3 = 50; 4 = 75; 5 = 100) for some analyses.

#### Clinical measures

Two clinical assessments, FEV1 and the 6MWT, were obtained from participants in a stable state (either baseline or week 12). FEV1 was measured using a portable spirometer administered by a research assistant trained in spirometry. Study-related spirometry was not performed if the patient had already undergone testing in-clinic that same day. The 6MWT measured the distance in meters that a participant was able to walk in a six-minute time span [67].

### Data analysis

#### Sample size considerations

Sample size estimates were based on an ability to detect approximately medium effect sizes (ES) in PROMIS scores between stable and acute COPD patients during the 12-week period [68–70]. With intraclass correlation of 0.5, a sample size of 81 in each group would enable us to detect a medium between-group ES with 80% power when using a two-sided alpha level of 0.05. Based on our previous research experience and the literature, we anticipated a 10% attrition rate over the 12-week study period. Thus, we targeted a total sample size of 180 based on a 10% attrition rate to account for potential study dropout and missing data for the planned study. All power calculations are based on the methods recommended in Cohen [71] and Kraemer and Thieman [72].

Data was summarized using descriptive statistics (e.g., means and standard deviation for continuous (and ordered) variables; frequency, mode and percentages for categorical variables) for demographic variables and all PRO and clinical measures. All item responses were examined using measures of central tendency (mean, median), spread (standard deviation, range), and response category (frequencies).

Responsiveness is an aspect of construct validity [73] and is estimated by evaluating the relationship between changes in clinical or patient-reported “anchors” and changes in the PRO scores over time; it can be evaluated in intervention studies, clinical trials and observational studies [74]. The COnsensus-based Standards for the selection of health Measurement Instruments (COSMIN) initiative [75] has proposed a definition of “responsiveness” as “The ability of an HR-PRO instrument to detect change over time in the construct to be measured.” In this paper, we examine responsiveness of the PROMIS measures over 12 weeks in patients with COPD who were recovering from an acute COPD exacerbation.

As detailed in Table 1, we hypothesized that some PROMIS domains (physical function, fatigue, anxiety, dyspnea severity, dyspnea functional limitations, discretionary social activities and social roles) would be impaired during a COPD exacerbation, and recovery from the exacerbation would be associated with improvements in these domains. Associated with changes in these physical and mental health domains, we also hypothesized responsiveness during recovery in Global Health (physical health and mental health). We hypothesized no or small changes over the 12 week recovery period for depression, anger, pain interference, and pain behavior. We hypothesized little change between baseline and 12 weeks on domains reported by participants enrolled in a stable state, and any change observed would be of a lesser magnitude than that observed in the exacerbation group. Thus, we compared the slope of changes in PROMIS and other PRO scores using mixed models (details below). We used Cohen’s *f*^2^ as a measure of local effect within a multivariate, mixed-effects regression model [71]. Cohen’s *f*^2^ convention is to classify these ES as small (≥0.02), medium (≥0.15) or large (≥0.35).

**Table 1:** PROMIS Measures and Hypothesized Responsiveness in COPD, with Results

PROMIS Measure	Hypothesized Responsiveness	Hypothesized Magnitude of Change	Results (responsive/magnitude)
Physical Function	Yes	Large	No/no effect
Pain Interference	No	Small	No/no effect
Pain Behavior	No	Small	No/no effect
Fatigue	Yes	Large	Yes/small
Anxiety	Yes	Medium	Yes/small
Depression	No	Small	Yes/trivial
Anger	No	Small	No/no effect
Discretionary Social Activities	Yes	Large	No/trivial
Social Roles	Yes	Large	Yes/small
Dyspnea - Severity	Yes	Large	No/trivial
Dyspnea - Functional Limitations	Yes	Large	No/trivial
Global Health - Physical	Yes	Large	Yes/no effect
Global Health - Mental	Yes	Medium	No/no effect

The mixed-effect model approach provides the flexibility of modeling not only the means of the data (as in the standard linear model), but also individual patients’ variation over time. Mixed models take into account the fact that measurements taken close in time are more highly correlated than measurements taken far apart in time. With mixed effect model approach, the slope of change for fixed effect (e.g., between baseline and 12-weeks later) is equivalent to the average group change. Other advantages of using mixed models include its handling of unbalanced research designs and missing data. In model fitting, the mixed model uses all available data from each subject rather than only including data from the subjects who have complete data at all of the time points. While we attempted to over-sample to compensate for the foreseeable loss of subjects due to dropout, the use of mixed models minimizes the impact of data missing throughout the course of the study. We hypothesized that there would be significant improvement (positive slope) in most PROMIS and clinical measures in COPD patients during recovery to a stable period. For each PROMIS and clinical outcome measure, the mixed model included group (fixed effect; i.e., stable and exacerbation), time (fixed effect; i.e., baseline and 12 weeks), and group by time interaction. We anticipated there would be significant group by time interaction effects in the PRO measures hypothesized to show large magnitude change (i.e., physical function, fatigue, anxiety, dyspnea severity, dyspnea functional limitations, discretionary social activities, social roles, Global physical health and Global mental health), illustrating a different slope of change between the stable and exacerbation groups across the 12-week study period for these domains.

Product-moment correlations between clinical measures (6MWT and FEV1) and PROMIS and SGRQ scores were estimated at baseline for stable patients and for patients enrolled in an exacerbated state, when the patients were deemed stable (i.e., 12 weeks after the baseline visit).

## Results

Across four sites, 770 individuals were screened. Of those, 288 were ineligible (e.g., heart failure exacerbation, smoking status, altered mental status, etc.), 212 refused participation, 11 consented but withdrew before completing an assessment, and 74 were not enrolled for various and unknown reasons. One hundred individuals with COPD were enrolled in a stable state and 85 were enrolled in an exacerbated state. Of the stable subjects, 90 (90%) completed the study (3 withdrew due to health, 2 died, 3 lost to follow up, 2 withdrew for unknown reasons) and 11 were censored due to exacerbations. Of the 85 in the exacerbation group, 61 (72%) completed the study (2 withdrew due to health, 2 died, 11 lost to follow-up, 9 withdrew for unknown reasons), and 15 had subsequent exacerbations. Thus, at week 12 follow-up, there were 79 (79%) individuals remaining in the stable group and 46 (54%) in the exacerbation group.

We conducted post-hoc analyses to examine whether drop-out differed by group (exacerbation vs stable). Results of the binary logistic regression indicated that the exacerbation group experienced a higher rate of dropout than the stable group (χ^2^(1) = 12.5, *p* = .0004). We examined whether age, gender, education, physical function and fatigue were associated with dropout and found that only physical function approached significance (*p* = .0512). When we added physical function in the model, the drop-out rate difference between the groups remained significant (χ^2^(1) = 8.45, *p* = .0037). There were no significant group by covariate interactions.

Baseline demographic and clinical characteristics of the enrolled subjects are presented in Table 2. Those enrolled in stable and exacerbation states did not differ on any demographic characteristic other than age, with the exacerbation group patients being younger. Exacerbation group participants also reported worse COPD and health at baseline. More patients enrolled in an exacerbation state reported having a COPD diagnosis for less than a year (*p* = .03), having more exacerbations in the past 12 months (80%, *p* < .0001), more COPD-related hospitalizations in the past 12 months (72%, *p* < .0001) and more COPD-related ED visits in the past 12 months (50%, *p* < .0001).

**Table 2:** Baseline Demographic and Clinical Characteristics of Enrolled Patients

Characteristic	Stable (n = 100)	Exacerbation (n = 85)	*p*-value
	mean (sd) range	mean (sd) range	< 0.01
Age	64.6 (9.3)	60.5 (10.2)	
	Range: 43–85	Range: 41–88	
BMI	31.10 (8.5)	32.10 (11.1)	0.51
	Range: 18–65	Range: 16–79	
Smoking pack year history	46.68 (27.3)	48.99 (34.5)	0.62
	Range: 10–156	Range: 10–180	
	n (%)	n (%)	*p*-value
Age Category			< 0.01
40–49 years	2 (2)	8 (9)	
50–59 years	28 (28)	37 (44)	
60–69 years	37 (37)	25 (29)	
70+ years	33 (33)	15 (18)	
Gender (Female)	43 (43)	43 (51)	0.30
Race (White)	75 (75)	60 (73)	0.78
Percent predicted FEV1			0.41
> = 80	4 (5)	2 (3)	
50–79	35 (42)	27 (39)	
30–49	33 (39)	23 (33)	
< 30	12 (14)	17 (25)	
6 min walk (> 300 m)	46 (55)	17 (43)	0.20
Length of COPD diagnosis			0.03
< 1 yr	4 (4)	14 (17)	
1–3 yrs	23 (23)	20 (24)	
3–5 yrs	15 (15)	14 (17)	
> 5 yrs	56 (57)	36 (43)	
Exacerbations last 12 months			< 0.0001
0	67 (68)	6 (7)	
1	17 (17)	24 (28)	
2 to 5	13 (13)	46 (54)	
> 6	2 (2)	9 (11)	
COPD hospitalizations last 12 months			< 0.0001
0	84 (87)	13 (15)	
1	9 (9)	39 (46)	
2 to 5	4 (4)	30 (36)	
> 6	0 (0)	2 (2)	
COPD ED visits last 12 months			< 0.0001
0	81 (83)	35 (41)	
1	9 (9)	23 (27)	
2 to 5	7 (7)	25 (29)	
> 6	1 (1)	2 (2)	
COPD medications^a^			
Antibiotics	1 (1)	20 (24)	< 0.0001
Beta agonists	94 (96)	80 (96)	0.87
Inhaled steroids	59 (60)	52 (63)	0.74
Systemic steroids	3 (3)	40 (48)	< 0.0001
Anticholinergenics	72 (74)	64 (77)	0.65
GOLD classification			0.43
GOLD 1 mild COPD	18 (19)	12 (17)	
GOLD 2 moderate COPD	36 (39)	24 (33)	
GOLD 3 severe COPD	25 (27)	28 (39)	
GOLD 4 very severe COPD	14 (15)	8 (11)	

^a^Responses are not mutually exclusive; participants could select more than one response

The PRO scores for the baseline and 12-week follow-up by baseline enrollment status as well as the responsiveness in PRO measures over 12 weeks, as evaluated by the difference between stable and exacerbation group differences over 12 weeks is summarized in Table 1 and detailed in Table 3. Significant differences were found in anxiety SF/CAT (*p =* .001/*p* < .01), fatigue (*p* < .0001/*p* < .0001), social roles (*p* < .001/*p* < .05) and depression (*p* < .05, SF only), with the exacerbation group reporting greater change (improvement) than the stable group. The magnitude of change (ES) for SF/CAT anxiety (0.023/0.021), fatigue (0.036/0.047) and social roles (0.035/0.024), which were hypothesized to be medium/large/large, respectively, were all small. The ES for depression (0.018) was trivial (less than the threshold for small). Physical function, discretionary social activities, and the Global-physical and Global-mental health scales, all hypothesized to improve during recovery, did not significantly change from baseline and had no to trivial ES. Dyspnea severity and dyspnea functional limitations, also hypothesized to improve during recovery, did not change significantly but showed near-small ES (0.017, 0.015, respectively). All other PROMIS domains that were hypothesized to have small to large ES demonstrated trivial to no effect sizes. The SGRQ symptoms, impacts and total scores (but not activities) also showed significant change from baseline (all *p* < .0001) and all (but activities) demonstrated small effect sizes.

**Table 3:** Changes in patient-reported outcomes (PRO) scores from mixed model^1^ (*N* = 185)

Measure	Stable Group		Exacerbation Group		Difference in Differences	F-statistic	*P*-value	f^2^ Effect Size^c^	95% CI
	Baseline Mean^b^	12 week Mean^b^	Baseline Mean^b^	12 week Mean^b^					
PROMIS Short Forms									
Anger	51.41	50.24	50.96	49.86	0.07	0.00	0.96	0.000	[−0.001, 0.001]
Anxiety	53.99	51.25	58.11	54.30	−1.06	3.18	< 0.001	0.023	[0.022, 0.024]
Depression	51.84	50.51	54.63	53.16	−0.13	2.38	< 0.05	0.018	[0.017, 0.019]
Fatigue	55.37	54.75	61.63	56.97	−4.04	4.89	< 0.0001	0.036	[0.035, 0.037]
Pain Behavior	53.63	52.12	55.54	53.63	−0.39	0.06	0.80	0.000	[−0.001, 0.001]
Pain Interference	57.29	55.66	59.43	57.26	−0.53	0.15	0.70	0.001	[0.000, 0.002]
Physical Function	36.97	37.29	34.50	35.35	0.54	1.41	0.17	0.011	[0.011, 0.011]
Discretionary Social Activities	45.18	45.93	42.74	45.27	1.79	1.45	0.21	0.011	[0.010, 0.012]
Social Roles	43.26	42.51	39.38	42.27	3.64	4.73	< 0.001	0.035	[0.034, 0.036]
Dyspnea Severity	51.15	50.69	56.70	54.22	−2.01	2.68	0.10	0.017	[0.015, 0.019]
Functional Limitations	50.36	50.33	53.86	52.14	−1.69	1.77	0.18	0.015	[0.013, 0.017]
Global Physical	39.20	39.56	35.98	37.60	1.26	1.86	0.17	0.008	[0.007, 0.009]
Global Mental	43.75	43.07	45.02	44.71	0.37	0.12	0.73	0.001	[0.000, 0.002]
PROMIS CATs									
Anger	52.11	50.86	51.46	50.21	0.01	0.00	0.99	0.000	[−0.001, 0.001]
Anxiety	54.99	52.13	59.64	55.42	−1.36	2.75	< 0.01	0.021	[0.021, 0.021]
Depression	51.98	50.32	55.27	53.50	−0.12	1.62	0.17	0.012	[0.011, 0.013]
Fatigue	56.80	54.53	63.24	57.08	−3.89	6.26	< 0.0001	0.047	[0.046, 0.048]
Pain Behavior	53.98	52.43	55.90	54.43	0.08	0.00	0.96	0.000	[−0.001, 0.001]
Pain Interference	57.53	55.51	60.72	58.18	−0.51	0.10	0.76	0.001	[0.000, 0.002]
Physical Function	37.11	37.34	35.26	35.78	0.29	0.98	0.46	0.007	[0.007, 0.007]
Discretionary Social Activities	44.33	45.57	41.99	44.91	1.68	1.19	0.31	0.009	[0.008, 0.010]
Social Roles	42.89	42.61	39.54	42.31	3.05	3.03	0.02	0.024	[0.023, 0.025]
SGRQ									
SGRQ Symptoms	57.24	58.30	71.74	58.36	−14.44	24.54	< 0.0001	0.133	[0.129, 0.137]
SGRQ Activities	68.61	67.09	77.35	72.26	−3.57	2.03	0.15	0.011	[0.009, 0.013]
SGRQ Impacts	40.66	40.15	53.25	43.18	−9.56	15.31	< 0.001	0.094	[0.090, 0.098]
SGRQ Total	51.84	51.30	63.66	54.50	−8.62	18.98	< 0.0001	0.118	[0.114, 0.122]
MMRC	41.28	37.85	53.04	48.95	−0.66	0.02	0.89	0.000	[−0.001, 0.001]

^a^Results in this table come from a mixed-effects model that included age, number of exacerbations in past year, group (fixed effect; i.e., stable or exacerbation), time (fixed effect; i.e., baseline or 12 weeks) and group-by-time interaction; ^b^least square means; ^c^Cohen f^2^, ≥0.02 = small, ≥0.15 = medium, ≥0.35 large; *CAT* Computer adaptive test, *SGRQ* St. George’s Respiratory Questionnaire, *MMRC* Modified Medical Research Council dyspnea scale

As a reminder, the 6MWTs on patients enrolled in an exacerbated state were performed at the 12-week follow-up when patients had returned to stable state. Six-minute walk scores were most highly correlated with PROMIS CAT scores for physical function (*r* = 0.50, p < .0001), fatigue (*r* = − 0.29, *p* < .01), depression (*r* = − 0.26, p < .01), discretionary social activities (*r* = 0.32, p < .01) and social roles (*r* = 0.32, p < .01), with similar correlations with SFs. Dyspnea severity/functional limitations SFs (*r* = − 0.39/− 0.35, p < .01) and SGRQ Impacts (*r* = − 0.22, p < .01) were also associated with six-minute walk scores. Percent predicted FEV1 scores were significantly correlated with PROMIS CATs for physical function (*r* = 0.32, p < .01), dyspnea severity (*r* = − 0.39, p < .0001), functional limitations (*r* = − 0.25, p < .01) and SGRQ Activities (*r* = − 0.25, p < .01).

## Discussion

The HRQL of COPD patients during an exacerbation is known to be significantly poorer than COPD patients in a stable state [16, 41, 68, 76], and in our study, participants enrolled during an exacerbation reported worse baseline health than those enrolled in stable status on nearly all HRQL measures. Given the significant symptom burden associated with exacerbations, these known group differences were expected and provide some evidence of construct validity of some PROMIS measures for people with COPD.

Of the nine domains hypothesized to be responsive to recovery from an exacerbation, only three demonstrated statistically significant change using both SF and CAT, and in none of the three was the hypothesized magnitude of change supported by the findings; all demonstrated small effect sizes. We had hypothesized that pain interference, pain behavior, depression and anger would not change over the 12 weeks. Only the change in the depression SF (not CAT) was statistically significant and the ES was trivial (below the threshold for small). Finally, we hypothesized that stable participants (exacerbation-free at enrollment and throughout the study period) would not demonstrate any change in PROs from baseline to the 12-week follow-up. The changes in PRO scores over 12 weeks in the stable group did reflect some change, but the changes were uniformly of lesser magnitude than the changes in the exacerbation group. Given the stability in their COPD during this time, such changes may reflect variations in other life and non-measured health events in these patients.

While some of the findings were unexpected, the most curious and unexpected finding was the lack of change in PROMIS physical function. Of note, the SGRQ did not demonstrate change in the activity section of the instrument, which is most similar to the PROMIS physical function. One possibility is that physical function does not change during recovery from an exacerbation, but we reject this notion based on obvious clinical characteristics of these patients. Declines in physical function with the onset of exacerbations are well documented [48, 77, 78]. Furthermore, physical function (as measured with PROMIS) has been shown to affect people across the spectrum of disease severity, including mild disease, whereas mental health was impaired only in patients with more severe disease [78]. Lung function does not always return to pre-exacerbation levels following an exacerbation, but the trajectory of recovery of physical function/activity is less well established [79–81]. Some reports have documented recovery to (or nearly to) baseline levels [82]. One of the few longitudinal studies evaluating “objectively” measured (accelerometer) physical activity for up to 6 months during COPD exacerbations and periods of clinical stability found that physical activity decreased significantly during exacerbations and persisted for about 2 weeks after symptomatic recovery. Others have reported a slow recovery trajectory or failure to return to baseline [5, 83], leading some to suggest that recovery in health status after an exacerbation may take longer than previously expected [48]. It is possible that improvement in physical function following an exacerbation takes longer than 12 weeks. In addition to the literature that contradicts this [81, 84, 85], we censored individuals with subsequent exacerbations, which is likely to have eliminated those with an extended recovery trajectory. Finally, these patients may be sedentary in the absence of exacerbation and not regularly testing their own physical function [86, 87].

An alternative explanation is that physical recovery occurs, but the PROMIS physical function measures (and SGRQ activity measure) did not detect it. This might have occurred for several reasons. The severity of exacerbations experienced by our participants might not have been of sufficient magnitude to be reflected in physical function recovery; however, 38 of the participants enrolled during an exacerbated state were hospitalized for their exacerbation, suggesting severe exacerbations. It is also possible that the sample size limited the power to detect significant change. The dropout in the exacerbation group was larger than anticipated and resulted in a sample size at follow-up smaller than the power analysis indicated was needed to detect hypothesized change. However, some other domain measures demonstrated significant change, so there appeared to be adequate power for the other domains. Another explanation is the possibility that, for the physical function items, patient responses during an exacerbation did not reflect their true current (i.e., exacerbation) state. Most PROMIS domains have a 7-day context (“In the past 7 days …”), but the physical function items do not have a specific time interval. It is possible that patients were not reflecting on their exacerbation (and often hospitalized) state, but rather they were considering their physical capability prior to deterioration. Thus, the lack of a context might represent an opportunity for participants to variably interpret their “current” state of health. The lack of context may work well in a state without rapid changes (e.g., arthritis), but may not work as well for conditions with acute periods of worsening (e.g., COPD). Some support for this derives from analyses of other chronic conditions. For example, the PROMIS physical function scores for stable patients and those recovering from a COPD exacerbation in this study (34–38) are not altogether dissimilar from physical function scores from samples of patients with back pain and chronic heart failure prior to receiving an intervention (38 and 35, respectively) [36]. However, the back pain and heart failure samples demonstrated notable improvements in physical function scores in response to clinical interventions (all *p* ≤ 0.001) [36]. Investigators hypothesized that, in contrast with the relatively stable disability associated with back pain and heart failure, acute worsening associated with COPD exacerbations may lead patients to over-report their physical function because they reference their usual state rather than acutely ill state [36]. However, to our knowledge, there is no evidence at present to support the possibility that reframing the question (e.g., “Considering how you feel right now…”) would produce different results, so this remains a hypothesis for future research to evaluate.

Similarly, we did not demonstrate significant change in the PROMIS dyspnea severity and functional limitation measures. It’s reasonable to expect dyspnea severity and functional limitations to improve during recovery from a COPD exacerbation, as dyspnea is one of the primary symptoms associated with an exacerbation. However, these were newly developed measures and this was the first longitudinal study in which they had been administered. Items on both measures reference the same set of activities, and individuals may not have had the opportunity to perform some of these activities during an exacerbation state, especially if hospitalized (e.g., preparing meals, washing dishes). The MMRC dyspnea scale score also did not improve during the recovery, and it is similarly based on activity (e.g., walking, strenuous exercise, etc.). Although the effect sizes reflecting change in PROMIS dyspnea severity and functional limitations approached the threshold for small magnitude and did not reach statistical significance in this study, prior cross-sectional studies have provided support for the reliability and validity of these measures in COPD [62, 63, 78] and other chronic lung diseases [88], suggesting that the small sample size might have limited the responsiveness observed here.

Our findings indicate that responsiveness to change was demonstrated for three of the nine PROMIS domains in which change was hypothesized. While there is only limited support for responsiveness among the domains tested, the score differences and the effect sizes reflecting the difference in differences between the two groups reflected greater magnitude of change in the exacerbation group. With the exception of depression, responsiveness to changes was similar for the PROMIS dynamic CATs and corresponding static SFs, suggesting there is no advantage of one administration option over the other, with equivalent precision and responsiveness to clinicians and researchers. CAT administration offers the advantage of minimal participant burden without sacrificing measurement precision, but requires a computer for administration. Short forms can be administered via paper and pencil and do not require a computer for administration. Both were developed with rigorous qualitative and quantitative methodology and offer the advantages of comparability across conditions, reliability, validity, and precision.

SGRQ total, symptoms, and impacts scores significantly discriminated longitudinal change between the stable and exacerbation groups, indicating the health status of these two groups were different, consistent with the intent of the study design. Three PROMIS measures fatigue, anxiety and social roles, also demonstrated differential longitudinal change between the two groups. We note also that the magnitude of effect sizes for the SGRQ symptoms and impacts subscales scores and total score were in the small range, albeit larger than that of the PROMIS measures. This is not unexpected, because one of the putative benefits of disease-specific compared to generic measures is their sensitivity to the disease itself. The SGRQ mean score for symptoms, impacts and total score (but not activities) for the exacerbation group also exceeded the minimal clinically important difference threshold estimates reported in the literature (4–7), including the higher threshold (7+) for patients with severe disease [89–93].

There are several limitations to this study worth noting. Both groups experienced drop-out, but this was most prominent in the exacerbation group, which reduced the available sample size and precision of our estimates of change. However, we were still able to demonstrate responsiveness, i.e., change over the course of recovery from an exacerbation, in some measures that were hypothesized to reflect such a change. Nevertheless, the high dropout rate likely limited our power to detect the hypothesized effect size. The demographic and clinical characteristics of patients lost to follow-up were not significantly different from those of patients that completed the study. Fatigue and physical function (as measured by both PROMIS SF and CAT) were associated with drop out, however, with those who dropped out from the exacerbation group demonstrating the highest levels of fatigue and lowest levels of physical function. The findings still showed strong evidence of responsiveness in the fatigue measures in the exacerbation group, but it is unclear if or how this might have impacted responsiveness of the physical function measures. We recruited participants across four distinct clinical sites, but our sample size precluded our ability to analyze site differences. However, a rigorous three-day face-to-face training was held for all study staff before the study was launched to standardize implementation of the study protocol and ensure consistent recruitment, enrollment and assessment procedures.

## Conclusion

This longitudinal study provides some initial support for the responsiveness of the PROMIS fatigue, anxiety and social roles domains relevant to COPD. As responsiveness is one element of a measure’s validity [73], this study provides some preliminary evidence of validity of some of the PROMIS measures as well. Further longitudinal studies with larger sample sizes are required to evaluate additional aspects of reliability and validity of the PROMIS measures and their performance in the COPD population. Additional evaluation of selected domains, especially the PROMIS physical function domain, is warranted, given the lack of responsiveness in this study.

Because PROMIS measures are generic, they can be used to assess the relative health status of respondents across diseases or functional characteristics. The multiple options for PROMIS administration (various short form lengths and CAT), allow for a customizable and efficient solution for measuring health outcomes important to patients. PROMIS is a useful tool for tracking select domains of HRQL in patients with COPD for research and possibly for clinical care.

## Data Availability

The datasets generated and/or analyzed during the current study are available for download from HealthMeasures Dataverse on the HealthMeasures website at: http://www.healthmeasures.net/resource-center/measurement-science/datasets-for-your-research.

## Abbreviations

6MWT: 6 min walk test
ADL: Activities of daily living
ANOVA: Analysis of variance
BMI: Body mass index
CAT: Computer adaptive test
COPD: Chronic obstructive pulmonary disease
COSMIN: COnsensus-based Standards for the selection of health Measurement Instruments
ED: Emergency department
FACIT: Functional Assessment of Chronic Illness Therapy
FEV1: Forced expiratory volume in 1 s
FVC: Forced vital capacity
HRQL: Health-related quality of life
IRB: Institutional review board
MMRC: Modified Medical Research Council dyspnea scale
PRO: Patient-reported outcome
PROMIS: Patient-Reported Outcomes Measurement Information System
SGRQ: St. George’s Respiratory Questionnaire
